# Patterns of local-regional failure after primary intensity modulated radiotherapy for nasopharyngeal carcinoma

**DOI:** 10.1186/1748-717X-9-60

**Published:** 2014-02-19

**Authors:** Fangfang Kong, Hongmei Ying, Chengrun Du, Shuang Huang, Junjun Zhou, Junchao Chen, Lining Sun, Xiaohui Chen, Chaosu Hu

**Affiliations:** 1 Department of Radiation Oncology, Fudan University Shanghai Cancer Center, 270 Dong’an Road, Shanghai 200032, P.R China; 2 Department of Oncology, Shanghai Medical College, Fudan University, Shanghai 20032, P.R China

**Keywords:** Intensity-modulated radiotherapy, Patterns of local-regional failure, Nasopharyngeal carcinoma

## Abstract

**Background:**

To analyze patterns of local-regional failure after primary intensity modulated radiotherapy (IMRT) for nasopharyngeal carcinoma (NPC).

**Methods:**

A total of 370 non-metastatic NPC patients consecutively treated with IMRT (with or without chemotherapy) were analyzed. Radiotherapy was administered using a simultaneous integrated boost (SIB) technique at the total prescribed dose of 66-70.4Gy (2.0-2.2Gy per fraction). The location and extent of local-regional failures were transferred to the pretreatment planning computed tomography (CT) for dosimetric analysis. The dose of radiation received by V_recur_ (volume of recurrence) was calculated and analyzed with dose-volume histogram (DVH). Failures were classified as: "in field" if 95% of V_recur_ was within the 95% isodose, "marginal" if 20% to 95% of V_recur_ was within the 95% isodose, or "outside" if less than 20% of V_recur_ was inside the 95% isodose.

**Results:**

With a median follow up of 26 months, 25 local-regional failures were found in 18 patients. The 1- and 2-year actuarial local-regional control rates for all patients were 99.7% and 95.5% respectively. Among the 22 local–regional failures with available diagnostic images, 16 (64%) occurred within the 95% isodose lines and were considered in-field failures; 3 (12%) were marginal and 3 (12%) were outside-field failures.

**Conclusions:**

Intensity-modulated radiotherapy provides excellent local-regional control for NPC. In-field failures are the main patterns for local-regional recurrence. Reducing the coverage of critical adjacent tissues in CTV purposefully for potential subclinical diseases was worth of study. Great attention in all IMRT steps is necessary to reduce potential causes of marginal failures. More studies about radioresistance are needed to reduce in-field failures.

## Background

Intensity-modulated radiotherapy (IMRT) is a major breakthrough in the treatment of NPC [1]. It is capable of improving dose conformity for complex tumor targets and better protection for the adjacent organs. Encouraging results with IMRT have been consistently reported [1-15]. Kwong [16] and Su [4] reported a 3- and 5- year local control rate of 100% and 97.7% for early-stage NPC treated with IMRT alone. For locally advanced NPC, 2- and 5-year local control rate of 95.7% [12] and 94.9% [7] can be achieved after effective chemoradiotherapy.

However, distant metastasis and post-treatment relapse remain as the main causes for NPC deaths. In 2000, Dawson et al. [17] firstly demonstrated that the majority (75%) of local-regional relapses after IMRT as the primary treatment for head and neck cancer were in-field, in areas of previous disease which was judged to be at high risk at the time of RT planning. Their findings then motivate studies of dose escalation to the highest risk regions. Eleven years later, Ng et al. [1] reported similar failure patterns in NPC patients. Furthermore, they found that the locoregional failure rate was significantly related to the minimum target dose, and it was recommended to deliver at least 66.5Gy to the target volumes. A recent study by Orlandi et al. [18] showed that apart from the above factors, overall treatment time (OTT), patient positioning errors and anatomical changes during RT could also affect treatment results.

It is worthy of discussion because IMRT planning is usually associated with sharp dose gradients outside the target volumes; therefore, an inadequate definition of target volumes could increase the risk of geographic misses, which eventually lead to local-regional recurrence [18]. In addition, a case series reported by Cannon and Lee [19] showed that the risk of marginal miss may be increased when excessive parotid gland sparing was pursued.

The aim of this study is to analyze the local-regional failure patterns following IMRT for NPC in our institution. This analysis allows us to assess the adequacy and the overall quality of the treatment technique.

## Methods

### Patients and pretreatment evaluations

From December 2007 to April 2012, 370 newly diagnosed non-metastatic NPC patients treated by definitive IMRT in Shanghai Cancer Center of Fudan University were enrolled in this study. All patients underwent disease restaging using the AJCC 2010 staging system. This study was approved by the Institutional Review Boards of Fudan University Shanghai Cancer Center. Written informed consent was obtained from the patient for the publication of this report and any accompanying images.

### Intensity-modulate radiotherapy

#### Immobilization and simulation

Patients were immobilized in the supine position with a thermoplastic head and shoulder mask. Intravenous contrast-enhanced CT using slice thickness of 5 mm was performed for planning. Image fusion of the T1 sequences with gadolinium enhanced MRI was performed with the CT simulation images for target delineation. The CT data were imported to treatment planning system for treatment design.

#### Target delineation

The target volumes were defined in accordance with the International Commission on Radiation Units and Measurements Reports 50 and 62 [20,21]. The primary gross tumor volume (GTV_P) and involved lymph nodes (GTV_N) included all gross tumors was determined by imaging, clinical, and endoscopic findings. The enlarged retropharyngeal nodes were outlined together with primary GTV, as the GTV_P on the IMRT plans. For patients who received neoadjuvant chemotherapy, the pre-chemotherapy volume of the primary lesion was used for GTV-P delineation,and the post-chemotherapy volume of the lymph nodes was used for GTV-N delineation.

The clinical target volume (CTV) was defined as the GTV plus 5- to 10-mm margin to encompass any microscopic extension, together with the regional lymphatics. Two clinical target volumes (CTVs) were defined in our radiotherapy: CTV1 and CTV2. The CTV1 was defined as the high-risk region that included GTV_P plus 5- to 10-mm margin; CTV1 should also encompass the entire nasopharynx, skull base, parapharyngeal space, retropharyngeal lymph nodal regions, the anterior second of the clivus, inferior sphenoid sinus, pterygoid fossae, the posterior third of the nasal cavity and maxillary sinuses, and any high risk nodal regions, including the bilateral upper deep jugular nodes, and the near station of the positive lymph nodes. Neck levels IB (submandibular nodes) were selectively irradiated only if there was extensive nodal disease on the ipsilateral IIA/IIB region, extracapsular extension of the IIA lymph nodes, or tonsilla and lingual root involved by the primary tumor. The levels IA (submental nodes) were delineated only if the submandibular nodes or oral cavity were grossly involved by disease. The low-risk CTV (CTV2) referred to levels IV and Vb without metastatic cervical lymph nodes. There were two corresponding PTV_Cs in our radiotherapy: PTV_C1 (CTV1 +3 mm) and PTV_C2 (CTV2 +3 mm). The PTV_Cs would encompass the corresponding CTV with a 3-mm margin in all directions. However, when the CTV was near critical organs, such as the brainstem, spinal cord, PTV_C was generated as small as 1 mm.

The organs at risk (OAR) include the spinal cord, brain stem, optic chiasm, optic nerves, eyeballs, lens, temporal lobes, parotid glands, oral mucosa, larynx and temporomandibular joints. A 5-mm margin was added to the spinal cord and brainstem during optimization to form the planning organ-at-risk volume (PRV).

#### Treatment planning and delivery

All patients were treated with external-beam radiation therapy using 6-MV photons, 7-9 radiation fields. The treatment technique was simultaneous integrated boost (SIB) technique. The dose prescribed to PTV-G (GTV +5 mm) was 66Gy in 30 fractions for T1–T2 disease, 70.4Gy in 32 fractions for T3–T4 disease, and 66Gy in 30 or 32 fractions for lymph nodes involved by disease. The dose delivered to PTV-C1and PTV-C2 was 60Gy and 54Gy, respectively. All patients were treated one fraction per day, 5 days per week.

### Chemotherapy

About 92.2% patients received cisplatin based chemotherapy including neoadjuvant chemotherapy, concurrent chemotherapy and adjuvant chemotherapy. The most common regimen of neoadjuvant and adjuvant chemotherapy included two to three cycles of TP (docetaxel 60 mg/m^2^/day, day 1, cisplatin 25 mg/m^2^/day, days 1–3), TPF (docetaxel 60 mg/m^2^/day, day 1, cisplatin 25 mg/m^2^/day, days 1–3, and 5-fluorouracil 0.5 g/m^2^ /day, days 1–3), or GP (gemcitabine 1 g/m^2^/day, day 1, day 8, cisplatin 25 mg/m^2^/day, days 1–3) regimen. Induction chemotherapy was given every 3 weeks. Four weeks after the completion of RT, the adjuvant chemotherapy was administered every 3 weeks. Concurrent chemotherapy consisting of 80 mg/m^2^, days 1–3 of cisplatin, every 3 weeks for 2 to 3 cycles.

### Patient evaluation

All patients were evaluated weekly for treatment response and toxicity during radiation therapy. After IMRT, patients were clinically evaluated at predefined intervals, typically every 3 months in the first 2 years, every 6 months from the third year to the fifth year, and annually thereafter. Each follow-up included indirect mirror examination for the nasopharynx and palpation of neck nodes. MRI of the nasopharynx, chest CT scan, and ultrasound of abdomen were performed 3 months after the completion of IMRT and every 6–12 months thereafter. Additional tests were ordered when indicated to evaluate local or distant relapse.

### Definition of failure site

For patients with local-regional failure, the recurrent tumor volume (V_recur_) was identified on MRI scans or CT scans obtained at the time when recurrence was diagnosed and transferred to the pretreatment planning CT. The exact site and extent of each tumor were then compared with the pretreatment planning CT data sets, focusing on the 95% isodose lines. The dose of radiation received by V_recur_ was calculated and analyzed with dose-volume histogram (DVH). The failures were categorized as occurring inside or outside the high dose target volume, depending on the location of V_recur_: "in field" if 95% of V_recur_ was within the 95% isodose, "marginal" if 20% to 95% of V_recur_ was within the 95% isodose, or "outside" if less than 20% of V_recur_ was inside the 95% isodose [17].

### Statistical methods

The follow-up period was measured from the first day of treatment. The Statistical Package for Social Sciences (SPSS version 16.0) software was used for statistical analysis. Kaplan-Meier method was used to calculate the cumulative local failure-free survival (LFFS), regional failure- free survival (RFFS) and overall survival (OS).

## Results

### Rates of local-regional recurrence

A total of 370 patients were analyzed. Characteristics of patients, tumor stage and treatment factors were detailed in Table 1. The median follow-up was 26 months, with a range from 3 months to 62 months. The 1- and 2-year actuarial local-regional control rates for all patients were 99.7% (95% confidence interval [CI]: 100-99.1%) and 95.5% (95% CI: 93-98%) respectively. The 2-year LFFS, RFFS and OS were 97.7%, 97.0% and 94.1%, respectively.

**Table 1:** Patient characteristics and treatment factors (n = 370)

**Characteristic**	**Patients (%)**
Gender	
Male	273 (73.8)
Female	97 (26.2)
Age (yr)	
Median	50
Range	9–79
WHO histologic type	
I	3 (0.8)
II	76 (20.5)
III	285 (77)
Others^a^	6 (1.7)
Tumor classification	
T1-2	198 (53.5)
T3-4	172 (46.5)
Node classification	
N0-1	139 (37.5)
N2-3	231 (62.5)
Stage	
I/II	69 (17.2)
III/IV	301 (82.8)
Treatment factors	
IMRT treatment duration (days)	
Median (range)	45 (40–58)
RT alone	29 (7.8)
Chemotherapy	341 (92.2)

WHO World Health Organization, RT radiotherapy.

^a^Other WHO histologic type including nasopharyngeal adenoid cystic carcinoma, poorly differentiated adenocarcinoma, small cell carcinoma and carcinomatous change.

At their last follow-up visit, 18 patients (4.9%) developed clinical or radiographic local-regional recurrences. 78% (14/18) of the patients were locally advanced (staged T3 or T4). The median time from treatment to local-regional recurrence was 20.5 months (range 11 to 41 months). Twelve patients with a local-regional recurrence were successfully salvaged with surgery alone (4), chemotherapy alone (4), RT alone (1), neck dissection and RT (1), or RT and chemotherapy (2). The ultimate crude local regional control rate was 66.7%. For the six patients with local-regional recurrences that could not be salvaged, four patients suffered distant metastasis before, simultaneously, or shortly after local-regional recurrence and the other two refused to accept any treatment.

### Dosimetric data

Table 2 shows the DVH statistics for patients of local-regional recurrence. On average, the target volumes had excellent coverage and only 1.7% of the GTV_P and 0.3% of the GTV_N received <95% of the prescribed dose. The majority (91%-96%) of the GTV_P and GTV_N actually received more than 100% of the prescribed dose. A similar situation was found in CTV1 and CTV2. The mean dose to the CTV1 was 65.9Gy and 56.7Gy to the CTV2. Volume (%) receiving less than 95% of the prescribed dose was 0.7% to CTV1 and 0.4% to CTV2.

**Table 2:** Dose–volume histograms (DVHs) statistics for patients of local-regional recurrence

	**GTV_P**	**GTV_N**	**CTV1**	**CTV2**
	**Average (range)**	**Average (range)**	**Average (range)**	**Average (range)**
Volume (cc)	88.5 (35–140.3)	85 (34.2–167)	298.6 (117.8–530.8)	124 (27.1–228.1)
Dmax (Gy)	75.5 (71.7–77.6)	73.2 (71.2–75.3)	75.2 (70.4–77.6)	61.7 (58.9–63.9)
Dmean (Gy)	71.5 (68–72.9)	68.7 (68–69.9)	65.9 (62.1–69.2)	56.7 (55.5–57.5)
Dmin (Gy)	59.9 (52.7–65.4)	58.8 (48.4–64.2)	43.4 (17.5–54.5)	43.3 (7-51)
V95%	1.7 (0–6.3)	0.3 (0–1.5)	0.7 (0.2–2.1)	0.4 (0–1.4)
V100%	91.3 (77.3–98.9)	95.6 (90.9–98.9)	96.5(92.1–99.1)	96.6 (93.9–99.5)
V110%	0.06 (0–1.1)	0.7 (0–3.8)	44 (2.1–76)	5.7 (0–14.5)

GTV_P gross tumor volume of primary tumor, GTV_N gross tumor volume of involved lymph nodes, CTV1 clinical tumor volume of the high-risk region, CTV2 clinical tumor volume of lymph nodal regions at low risk. Dmax = Maximum dose, Dmean = Mean dose, Dmin = Minimum dose. V95% =%volume receiving < 95% of the prescribed dose, V100% =% volume receiving >100% of the prescribed dose, V110% =% volume receiving >110% of the prescribed dose.

### Patterns of failure

In the 18 patients who developed into local-regional recurrences, 7 patients had isolated regional recurrences, other 7 patients had isolated local recurrences, and 4 patients had both local and regional recurrences. 5 patients had simultaneous distant metastasis, including lung and bone metastasis.

A total of 25 recurrences were observed in the above 18 patients, 16 (64%) occurred within the 95% isodose lines and were considered in-field failures; 3(12%) were marginal, occurring in a steep dose gradient region at the margin of the high-dose PTV-C, the other 3 (12%) were outside-field failures. There were 3 missing values because of the unavailability of the diagnostic image. The sites of local-regional recurrences are detailed in Table 3.

**Table 3:** Details of recurrent patients and their local-regional failures

				**Dose–volume histograms statistics to recurrence volume**					
**No.**	**Stage**	**Site of relapse**	**Location of the recurrence volume**	**Vrecur (cc)**	**Dmean (Gy)**	**Dmin (Gy)**	**Dmax (Gy)**	**V**_**95% **_**(%)**	**Type of relapse**^**a**^
1	T4N3b	Local	CTV	13.9	69.9	48.5	75.2	97.9	In-field
		Regional	CTV	2.7	68.9	64.6	71.3	100	In-field
2	T4N1	Local	Marginal to CTV	10.5	70	51.5	77	94.3	marginal
3	T4N0	Local	CTV	32.9	70.9	37.6	75.4	97.7	In-field
4	T3N3b	Local	Marginal to CTV	13.9	62.1	11.8	74.1	74.7	marginal
		Regional	CTV	3.6	67.5	63.9	71.9	100	In-field
5	T2N1	Local	CTV	15.1	67.6	60	71.2	100	In-field
6	T1N0	Local	CTV	10.2	65.1	53.3	71.7	100	In-field
7	T3N3b	Local	CTV	2.2	70	63	76.8	100	In-field
		Regional	Outside CTV	8.4	14.2	11.2	37.7	-	outside
8	T3N1	Local	GTV	10.6	73.5	69.1	76.3	100	In-field
		Regional	CTV	5.5	69.6	65.9	73.6	100	In-field
9	T4N0	Local	GTV	9.3	72	61.8	74	99.2	In-field
10	T3N2	Local	CTV	15.2	71.9	63.3	77.3	100	In-field
11	T4N1	Local	CTV	8	72.1	66	73.8	100	In-field
12	T4N3b	Regional	-	-	-	-	-	-	-
13	T3N2	Regional	CTV	20.6	67.8	51.2	72.6	99.6	In-field
14	T2N1	Regional 1	Outside CTV	3.6	45.3	34.6	56.7	-	outside
		Regional 2	Marginal to CTV	12.6	53.4	20.6	66.7	32.5	marginal
		Regional 3	Outside CTV	2.7	54.2	46.6	61.4	19.1	outside
15	T3N2	Regional	-	-	-	-	-	-	-
16	T3N3b	Regional	CTV	4.2	66.5	61.3	69.3	100	In-field
17	T1N2	Regional	GTV	2.2	70.1	67.9	72.4	100	In-field
18	T3N3b	Regional 1	-	-	-	-	-	-	-
		Regional 2	CTV	22.2	65.6	51.4	73	98.8	In-field

CTV clinical target volume, GTV gross tumor volume; V95%=% of volume of failure receiving at least 95% of prescribed total dose, Vrecur the recurrent tumor volume.

^a^In-field refers to 95% of the recurrence volume receiving more than 95% of the prescribed dose. Marginal refers to 20–95% of the recurrence volume receiving 95% of the prescribed dose. Outside refers to less than 20% of the the recurrence volume receiving 95% of the prescribed dose.

**Figure 1:**
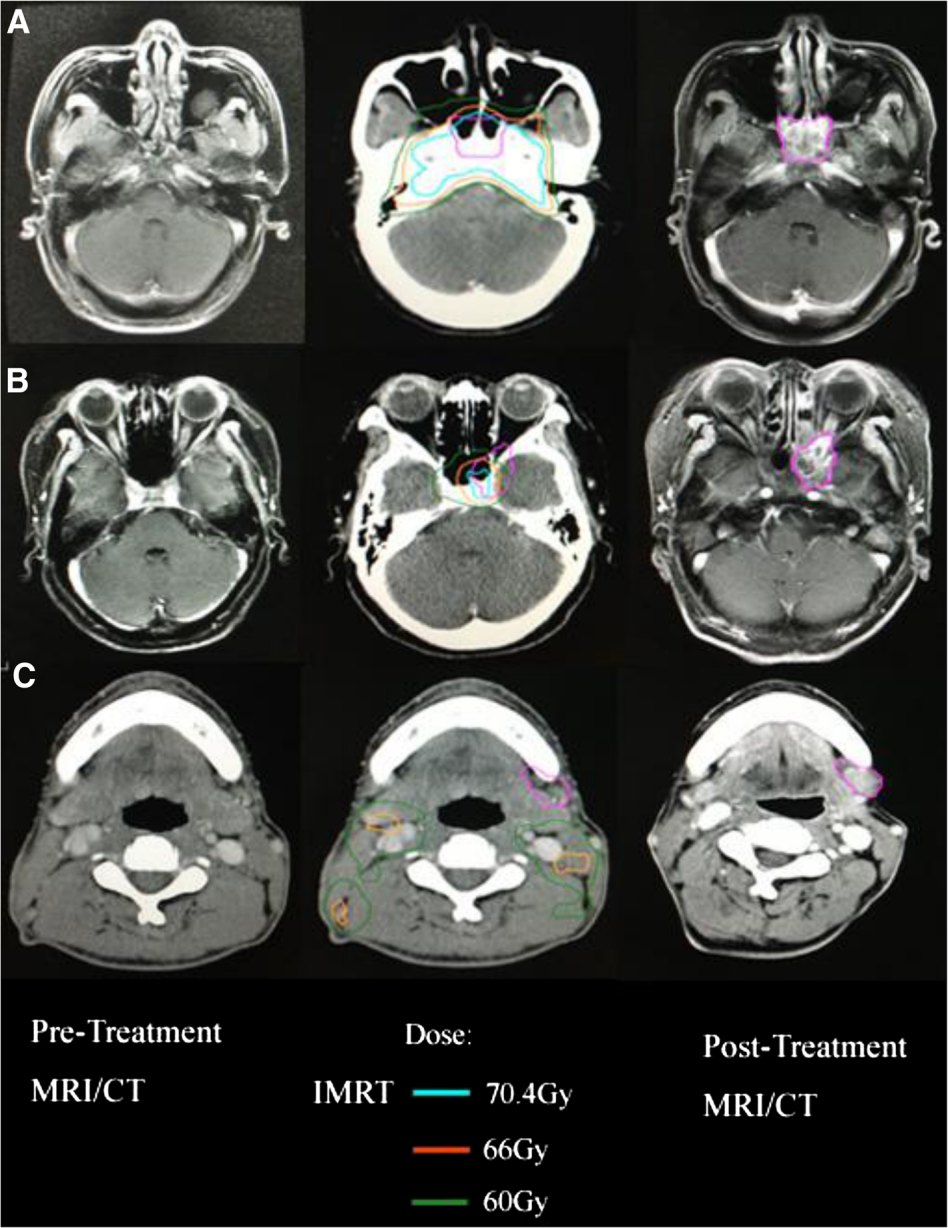
**Disease extent for patients having local-regional failures. A**, In-field failure. **B**, Marginal failure. **C**, Outside-field failure. Left, Pretreatment magnetic resonance imaging (MRI/CT). Middle, The recurrent tumor volumes were transferred from the diagnostic MRI/CT at the time of recurrence to the planning computed tomography to show doses delivered to the recurrence sites. Right, MRI/CT at time of failure. IMRT = intensity-modulated radiotherapy.

As show in Table 3, the average minimum, mean and maximum dose delivered to V_recur_ for marginal recurrence were 28Gy, 61.8Gy and 72.6Gy, respectively. Regarding the in-field recurrences, the average minimum dose to V_recur_ was 59.3Gy (range 37.6Gy to 69.1Gy). The average mean dose of radiation to the in-field recurrences was 69.3Gy (range 65.1Gy to 73.5Gy). The average maximum dose of radiation to the in-field recurrences was 73.5Gy (range 69.3Gy to 77.3Gy). Figure 1 demonstrates axial dose distributions through the epicenter of the recurrent volumes occurring in-field, marginal and outside-field.

## Discussion

There is little controversy that IMRT is the treatment of choice for NPC because dosimetric studies showed clear advantages by improving dose conformity for complex tumor targets and better protection of the adjacent organs [1]. The 1- and 2-year local-regional control rates of 99.7% and 95.5% in the present study are excellent and similar to reports from other centers [1-15].

As shown in Table 3, most of the relapses in our study occurred in locally advanced disease, within or marginally to the high dose region. Three out-field failures were observed in this study. Patient 7 (staged T3N3b) had treatment failure within a spared parotid gland. Parotid node involvement occurs in only 1% of cases [22]. This low proportion of patients does not justify the inclusion of this level in local-regional CTV. However, Cannon et al. [19] suggested that for patients who had multilevel nodal disease, including disease in level II, and underwent a neck dissection that could have contributed to modification of lymphatic drainage, parotid nodes could be considered for inclusion in local-regional CTV. Patient 14 (staged T2N1) suffered 3 times of regional recurrences one after another, two of them occurred in submandibular nodes. Neck levels IB (submandibular nodes) were selectively irradiated only if there was extensive nodal disease on the ipsilateral IIA/IIB region, extracapsular extension of the IIA lymph nodes, or tonsilla and lingual root involved by the primary tumor in our institution. The recent meta-analysis by Ho et al. reported that IB node involvement occured in only 3% of cases [22]. This low risk of involvement also supports the elective treatment of level IB. The 3^rd^ relapse occurred in neck levels IIA which grew alongside the space between the muscles, and then it was defined as "outside-field" failures. The potential reason is the neck dissections the patient subjected to after the previous relapses, which could have modified his lymphatic drainage.

It is generally recommended that a total dose of 70Gy with conventional fractionation should be given, but it is difficult to be administered without severe side effects in advanced T4 disease, even by IMRT [18]. Ng et al. [1] suggested a possible strategy to lessen the dose constraint criteria of selected neurologic structures (i.e. to sacrifice one side of the optic nerve or temporal lobe). In our study, patient 2 (stage T4N1) and patient 4 (stage T3N3b) developed cavernous sinus and orbital apex recurrence, which were relatively high locations in NPC. The possible reasons include missing of subclinical target volume and compromise with OAR. This also suggests the importance of cooperation between clinicians and radiologists. And great attention should be paid to the treatment precision for patients with adequate but very tight dose coverage to the target volumes, especially for patients with the very steep dose gradient proximal to the critical neurologic structures.

As show in our study, the main patterns of local-regional recurrence are in-field failures. A recent Medline review by Hong B et al. [23] showed that radioresistance may be the ultimate cause of a local-regional failure. They reviewed articles published on clinical and preclinical studies targeting tumor hypoxia and found that tumor hypoxia was common in NPC; it was associated with disease progression and resistance to therapy. In our series, 64% recurrences were located well within the 95% dose region. In these cases, it is reasonable to assume that there are nonuniform clonogenic cell density and radiosensitivity within the same target. Hence, we hope that with the development of radiation biology, a smarter, nonuniformly increased dose distribution can be established to reduce in-field failure as much as possible.

Despite of the prevailing use of IMRT in the treatment of NPC, optimal target volumes especially the clinical target volumes (CTVs) have not been sufficiently addressed. Our results suggest that the definition of CTVs currently used in our institution is sufficient. It provided excellent control in both the primary disease and involved neck areas. As reducing high-dose radiation to normal critical tissues is one of the major purposes of IMRT, it is questionable that if we can purposefully reduce the coverage of critical adjacent tissues in CTV for potential subclinical diseases. Lin et al. [24] found that IMRT using a reduced-volume technique did not increase incidence of local and /or regional recurrence that could be attributed to the reduction of clinical target volume adjacent to the primary disease. But further optimization and prospective researches are needed.

There were several limitations of this study. First, since the interval for enrollment was about 5 years, various factors such as radiation techniques, radiation doses and chemotherapy regimens have evolved. Second, due to the relatively small sample size and short time follow-up, the current findings could only be taken as preliminary. In order to illustrate patterns of local-regional failure and possible reasons following primary IMRT for NPC, longer follow-up and a large sample of uniform treatment are needed for further research.

In conclusion, our data shows excellent local-regional control by IMRT for NPC. The 1- and 2-year actuarial local-regional control rates for all patients were 99.7% and 95.5% respectively. In-field failures (64%) are the main patterns for local-regional recurrence. The occurrence of marginal failures implies that great attention should be paid in all IMRT steps to reduce potential causes of marginal failures. And studies focusing on radioresistant should be pursued to reduce in-field failures in the future. Reducing the coverage of critical adjacent tissues in CTV purposefully for potential subclinical diseases was worth of study.

## Abbreviations

IMRT: Intensity-modulated radiotherapy; NPC: Nasopharyngeal carcinoma; RLN: Retropharyngeal lymph node; DVH: Dose-volume histogram.

## Competing interests

The authors declare that they have no competing interests.

## Authors’ contributions

CD, SH and HY participated in the treatment panning, contributed to the data collection. HY, CH and FK participated in its design and coordination. JZ and XC conceived of the study and participated in the data collection. FK and HY performed the statistical analysis, and drafted the manuscript. All authors read and approved the final manuscript.
